# Using Topological Indices to Predict Anti-Alzheimer and Anti-Parasitic GSK-3 Inhibitors by Multi-Target QSAR *in Silico* Screening

**DOI:** 10.3390/molecules15085408

**Published:** 2010-08-09

**Authors:** Isela García, Yagamare Fall, Generosa Gómez

**Affiliations:** Department of Organic Chemistry, Faculty of Chemistry, University of Vigo, Spain

**Keywords:** glycogen synthase kinase-3 (GSK-3) inhibitors, Alzheimer’s disease, *Plasmodium falciparum*, *Trypanosoma brucei*, *Leishmania*, QSAR

## Abstract

*Plasmodium falciparum*, *Leishmania*, *Trypanosomes*, are the causers of diseases such as malaria, leishmaniasis and African trypanosomiasis that nowadays are the most serious parasitic health problems worldwide. The great number of deaths and the few drugs available against these parasites, make necessary the search for new drugs. Some of these antiparasitic drugs also are GSK-3 inhibitors. GSKI-3 are candidates to develop drugs for the treatment of Alzheimer’s disease. In this work topological descriptors for a large series of 3,370 active/non-active compounds were initially calculated with the ModesLab software. Linear Discriminant Analysis was used to fit the classification function and it predicts heterogeneous series of compounds like paullones, indirubins, meridians, *etc*. This study thus provided a general evaluation of these types of molecules.

## 1. Introduction

Neurofibrillary tangles (NFTs) are one of the characteristic neuropathological lesions of Alzheimer’s disease (AD) [1] and other neurodegenerative processes such as frontotemporal dementia, Pick’s disease, progressive supranuclear palsy, and corticobasal degeneration [2].

Alzheimer’s disease affects 5–10% of the population over 65 years of age. The dementia associated with AD results from the selective death of neurons, which is associated with several anatomo-pathological hallmarks such as senile neuritic plaques and neurofibrillary tangles [3,4]. Three molecular actors clearly play a role in the development of AD: the amyloid β peptide (Aβ), presenilins-1 and -2 and the microtubule-associated protein tau.

Glycogen Synthase Kinase-3 (GSK-3) is a senile-threonine kinase ubiquitously expressed and involved in the regulation of many cell functions [5]. GSK-3 was originally identified as one of the five protein kinases that phosphorylate glycogen synthase [6], being implicated in type-2 diabetes [7]. GSK-3 is also known to phosphorylate the microtubule-associated protein tau in mammalian cell [8]. This hyperphosphorylation is an early event in neurodegenerative conditions, such as Alzheimer’s disease [9], also involved is a second kinase, called CDK-5 [10].

Two GSK-3 genes (α and β) have been cloned from vertebrates. The intrinsic biochemical properties of GSK-3 are also conserved, with greater differences between GSK-3α and GSK-3β than between species [11].

Interest in GSK-3 has grown far beyond glycogen metabolism during the past decade and GSK-3 is known to occupy a central stage in many cellular and physiological events, including Wnt and Hedgehog signaling, transcription, insulin action, cell-division cycle, response to DNA damage, cell death, cell survival, patterning and axial orientation during development, differentiation, neural functions, circadian rhythm and others. In 1988 Ishiguro *et al*. [12] isolated one enzyme when they were studying an extract of the brain, which showed the generation of paired helical filaments of Tau protein kinase, typical injury of Alzheimer’s disease.

In this moment, there is an increasing interest in the evaluation of kinases from unicellular parasites and fungies as targets for potential new anti-parasitic drugs. The evolutionary difference between unicellular kinases and their human homologues might be sufficient to allow the design of parasite-specific inhibitors. The *Plasmodium falciparum* genome contains 65 genes that encode kinases, including three forms of GSK-3. *P. falciparum* is one of the *Plasmodium* species that cause malaria in humans. It is transmitted by the female *Anopheles* mosquito. Malaria has the highest rates of complications and mortality; for example in 2006 it accounted for 91% of all 247 million human malaria infections (98% in Africa) and 90% of the deaths.

*Leishmania* is a genus of trypanosome protozoa, and is the parasite responsible for the disease leishmaniasis. *Leishmania* commonly infects hyraxes, canids, rodents and humans; and currently affects 12 million people in the world. There are many drawbacks to current chemotherapy for leishmaniasis, including problems of low efficacy, severe toxic side effects, and emerging drug resistance [13,14,15].

*Leishmania* parasites posses a complex life cycle in which the parasite passes between the sandfly vector and the mammalian host, during which time the parasite oscillates between rapidly dividing and cell cycle-arrested forms. The cell cycle of *Leishmania* is closely regulated, as in other eukaryotes, and integrated with its differentiation between the various life cycle stages.

Trypanosomes are a group of kinetoplastic protozoa distinguished by having only a single flagellum. All members are exclusively parasitic, found primary in insects. A few genera have life-cycles involving a secondary host, which may be a vertebrate or a plant. These include several species that cause major diseases in humans. African Trypanosomiasis disease is caused by members of the *Trypanosma brucei* complex, is a serious health threat. It is estimated that 300,000 to 500,000 humans in sub-Saharan African are infected. Despite the critical need, the available therapies are becoming less satisfactory due to the rising level of resistance to the available drugs, the long period of treatment required to achieve a cure, and the unacceptable and sometimes severe adverse effects associated with current therapies [16]. However, optimization of the selectivity of drug candidates for parasite kinases becomes an issue due to the highly conserved amino acids and protein conformation of the catalytic domains [17,18,19,20]. Our main objective in this work was to identify numerous examples of anti-parasitic, anti-fungi, *etc.* and GSK-3 inhibitors in order to obtain a model for the optimization of selected target inhibitors for drug development [21,22].

There are more than one thousand theoretical descriptors available in the literature to represent molecular structures, and one usually faces the problem of selecting those which are the most representative for any particular property under consideration. Topological indices [23,24,25,26,27,28,29,30,31,32,33], the most commonly used molecular descriptors, have been widely used in the correlation of physicochemical properties of organic compounds. In chemical graph theory, molecular structures are normally represented as hydrogen depleted graphs, whose vertices and edges act as atoms and covalent bonds, respectively. Chemical structural formulas can be then assimilated to undirected and finite multigraphs with labeled vertices, commonly known as molecular graphs. Topological indices, also known as graph theoretical indices, are descriptors that characterize molecular graphs and contain a large amount of information about the molecule, including the numbers of hydrogen and non-hydrogen atoms bonded to each non-hydrogen atom, the details of the electronic structure of each atom, and the molecular structural features [34]. In this article, we use ModesLab software [35] for calculating Topological indices (see Table 1), which is very important in the area of development of molecular descriptors and its applications to quantitative structure-property (QSPR), quantitative structure-activity (QSAR) relationship and drug design. ModesLab also provides a very useful way to define the properties of atoms, bonds and fragments by an extension of SMILES language and use these properties in molecular descriptors calculations.

**Table 1:** Topological Indices used in the present study.

Index	Description
χ(P), χ(C), χ(PC), χ(Ch)	Randic branching index
χ_v_(P), χ_v_ (C), χ_v_ (PC), χ_v_ (Ch)	Valence connectivity
e(P), e(C), e(pC), e(Ch)	Epsilon index
^1^κ, ^2^κ, ^3^κ	Kappa index
^1^κ(alpha), ^2^κ(alpha), ^3^κ(alpha)	Kappa (alpha) index
Ø	Flexibility index
M_1_	Zagreb M_1_ index
M_2_	Zagreb M_2_ index
H	Harary number
J	Balaban index

The development of QSARs using simple molecular indices appears to be a promising alternative or complementary technique to drug-protein docking, high-throughput screening and combinatorial chemistry techniques. Almost all QSAR techniques are based on the use of molecular descriptors, which are numerical series that codify useful chemical information and enable correlations between statistical and biological properties [36,37,38].

A large number of examples have been published in which the use of molecular descriptors has become in a rational alternative to massive synthesis and screening of compounds in medicinal chemistry [39,40]. The principal deficiency in the use of some molecular indices concerns their lack of physical meaning.

## 2. Results and Discussion

### 2.1. General QSAR for GSK-3 Inhibitors

The development of a discriminant function [41] that allows the classification of organic compounds as active or non-active is the key step in the present approach for the discovery of GSK-3 inhibitors. It was therefore necessary to select a training data set of GSK-3 inhibitors containing wide structural variability. Linear Discriminant Analysis (LDA) was used to construct the classifiers. One of the most important steps in this work was the organization of the spreadsheet containing the raw data used as input for the LDA because this is not a classic classifier. Herein, the schematisation of the paper is peculiar. Our expectation is to use a two-group discriminant function to classify compounds into two possible groups: compounds that belong to a particular group and compounds that do not belong to this group. To this end, we have to indicate somehow what group we pretend to predict in each case. In this regard, we made the following steps, these steps are essentially the same given by Concu *et al.* [42,43] for their QSAR study of six classes of enzymes. In this study, each compound may be assayed on q^th^ different sets of conditions in the pharmacological tests, which are defined as Compound Assay Conditions query (CAC_q_). These conditions are indicated in the supplementary material. The selection here of discriminant techniques instead of regression techniques was determined by the lack of homogeneity in the conditions under which these values were measured. As reported in different sources, numerous IC_50 _values lie within a range rather than a single value. In other cases, the activity is not scored in terms of IC_50_ values but is quoted as inhibitory percentages at a given concentration. Once the training series had been designed, forward-stepwise Linear Discriminant Analysis (LDA) was carried out in order to derive the QSAR (see **Equation 1**):

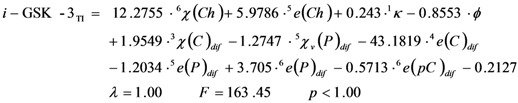
(1)


The statistical significance of this model was determined by examining Wilk’s λ statistic, Fisher ratio (F), and the p-level (p). The model is based on two types of parameters, the first type are parameters for single molecules. The type one includes first fourth parameters in the model. The following parameters presented in Table 1: randic branching index (χ(Ch), χ(C)), epsilon index (e(Ch), e(C), e(P), e(pC)), kappa index (κ), flexibility index (Ø) and valence connectivity (χ_v_(P)). In addition, we can see in the model parameters that quantify the difference between the structure of the drug and the structure of the drugs active for a given set of conditions CAC_q_ (see the last six parameters in the **Equation 1a-1f**). We quantify this information in terms of the difference between the descriptors (Ds) of the drug and the average of Ds of active drugs for a given condition (see Methods section). The exact formulas for these terms present in the model are:

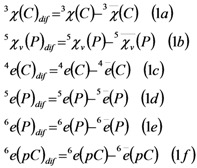



**Figure 1:**
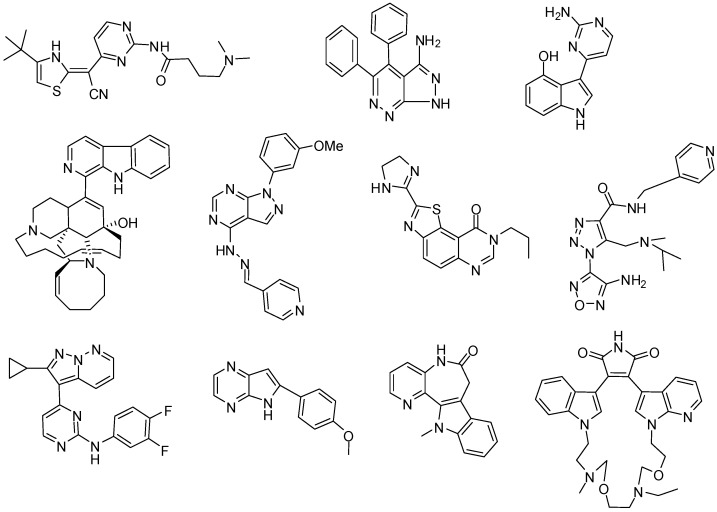
Some compounds studied in this article.

The introduction of this last parameter in the model (1) coincides with the i-GSK-3 activity observed in organic acid compounds [44] and drug connectivity may become an interesting alternative for fast computational pre-screening of large series of compounds in order to rationalize synthetic efforts [45,46,47,48,49,50,51] complementary to more elaborated techniques 3D-QSAR, CoMFA, and CoMSIA studies that depend on a detailed knowledge of 3D structure. In any case, these present models are of more general application than the other known methods that apply only to compounds tested in only one CAC and/or belonging to only one homogeneous structural class of compounds. Confirmation of this statement comes from the fact that the present classification function has given rise to an efficient separation of all compounds: with Accuracy = 99.1% in training series and Accuracy = 86.8% in validation series for the topological function, see Table 2 for details. The names, observed classification, predicted classification and subsequent probabilities for all 3,370 compounds in training and average validation are given as supplementary material. This level of total Accuracy, Sensitivity and Specificity is considered as excellent by other researches that have used LDA for QSAR studies and taking into account the great variety of compounds (see Figure 1), due to the fact that their structures are very different; see for instance the works of Garcia-Domenech, Prado-Prado and Marrero-Ponce *et al.* [52,53,54,55,56,57,58,59,60,61,62,63,64,65,66,67].

**Table 2:** Training and validation results.

Group	Parameter	%	GSKI-3	Non-active
			***Training***	
**GSKI-3**	*Sensitivity*	95.3	854	42
**Non-active**	*Specificity*	82.8	77	371
**Total**	*Accuracy*	91.1		
			***Validation***	
**GSKI-3**	*Sensitivity*	95.3	282	14
**Non-active**	*Specificity*	84.6	179	985
**Total**	*Accuracy*	86.8		

## 3. Materials and Methods

### 3.1. Computational Methods

**Figure 2:**
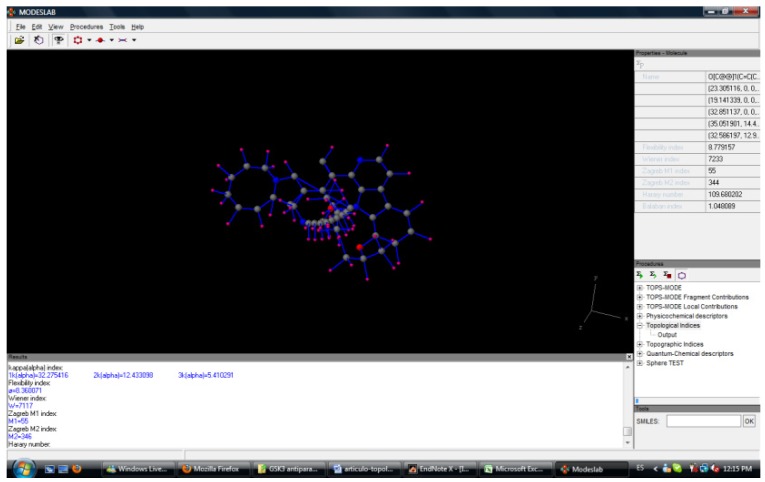
ModesLab software.

The dataset formed by 3,370 cases was divided into 43 groups depending of their GSK-3 inhibitory activity and antiparasitic, antifungi, *etc.* activity. The model was obtained with topological descriptors. ModesLab (Molecular Descriptor Laboratory) version 1.5 software [35] was used to calculate all descriptors (see Figure 2). 

The total analyzed variables were 189. The variables ^3^χ(Ch), ^4^χ(Ch), ^3^χ_v_(Ch), ^4^χ_v_(Ch), ^3^e(Ch), ^4^e(Ch), ^3^χ(Ch)_avg_, ^4^χ(Ch)_avg_, ^3^χ_v_(Ch)_avg_, ^4^χ_v_(Ch)_avg_, ^3^e(Ch)_avg_, ^4^e(Ch)_avg_, ^3^χ(Ch)_ dif_, ^4^χ(Ch)_dif_, ^3^χ_v_(Ch) _dif_, ^4^χ_v_(Ch)_dif_, ^3^e(Ch)_dif_, ^4^e(Ch)_dif_ was eliminated of the database because all of them were equal to 0 and constants. The quality of the model was determined by examining the Wilk’s statistic, the square of Mahalanobis distance (D^2^), the Fisher ratio (F) and the number of variables in the equation. Discrimination functions were obtained by using the forward-stepwise linear discriminant analysis as implemented in Statistica 6.0.

### 3.2. Multi-target Linear Discriminant Analysis (LDA)

In this regard, we performed the following steps, which are essentially the same given by Concu *et al.* [42,43] for their QSAR study of six classes of enzymes:

(1). We created a raw data representing each compound input as a vector made up of 1 output variable, 189 structural variables (inputs) divided in values (see the first term of the Equation (1)), averages (see the second term of the Equation (1)) and differences between values and averages (see the third term of the Equation (1)); and the CAC_q_ variable. CAC_q_ is an auxiliary not used to construct the model.
(2). The first element (output) is a dummy variable (Boolean) called Observed Group (OG); OG = 0 if the compound belongs to the class to which we refer in CAC_q_ and 1 otherwise (OG = 1). We could repeat each compound more than once in the raw data. In fact, we could repeat each compound 43 times corresponding to 43 CAC_q_ Assay Conditions (seeTable 3). The first time we used the CAC_q_ = CAC number. It means that we used the real CAC class of the compound in CAC_q_. In this case, the LDA model had to give the highest probability to the group OG = 0 because it had to predict the real class of the compound. The remnant 43 times we use an CAC class number different to the real in CAC_q_ and then the LDA model had to predict the highest probability for the group OG = 1. This indicated that the compound did not belong to this group.

**Table 3:** Compound Assay Conditions query (CACq).

*Group*	*Parameter*	*Enzyme*	*Isoform*	*Enzyme/ Organism*	*Species*	*Activity (1,0)*	*Condition*	*Observ.*
1	%	GSK-3	beta	enzyme	no	0	0	=
2	cKi	GSK-3	alfa	enzyme	no	0	100	>
3	EC_50 _(μM)	no	no	no	Cell Efficacy	0	2	>
4	EC_50_ (μM)	no	no	no	glycogen synthesis stimulation	0	inactive	=
5	EC_50_ (μM)	no	no	no	β-catenin synthesis	0	2	>
6	EC_50_ (μM)	no	no	parasite	*T. brucei*	0	2	>
7	EC_50_ (μM)	no	no	virus	HIV-1	0	NA	=
8	ED_50_ (μM)	no	no	parasite	*L. donovani*	0	5	<
9	IC_50_ (ng/mL)	no	no	parasite	*P. falciparum* (chloroquine resistant W2 clone)	0	NA	=
10	IC_50_ (ng/mL)	no	no	parasite	*P. falciparum* (chloroquine sensitive D6 clone)	0	NA	=
11	IC_50_ (nM)	GSK-3	alfa	enzyme	no	0	2000	>
12	IC_50_ (nM)	GSK-3	beta	enzyme	no	0	2000	>
13	IC_50_ (nM)	GSK-3	nd	enzyme	no	0	2000	>
14	IC_50_ (μg/mL)	no	no	bacterium	*M. intracellulare*	0	NA	=
15	IC_50_ (μg/mL)	no	no	bacterium	MRS	0	NA	=
16	IC_50_ (μg/mL)	no	no	bacterium	*S. aureus*	0	NA	=
17	IC_50_ (μg/mL)	no	no	cell line	Human Vero cells	0	NC	<>
18	IC_50 _(μg/mL)	no	no	fungus	*C. neoformans*	0	NA	=
19	IC_50_ (μg/mL)	no	no	parasite	*L. donovani*	0	NA	=
20	IC_50_ (μM)	GSK-3	beta	enzyme	no	0	2	>
21	IC_50_ (μM)	GSK-3	nd	enzyme	no	0	2	>
22	IC_50_ (μM)	GSK-3	no	parasite	*P. falciparum*	0	20	>
23	IC_50_ (μM)	GSK-3	α/β	enzyme	no	0	2	>
24	IC_50_ (μM)	no	no	bacterium	*M. intracellulare*	0	—	=
25	IC_50_ (μM)	no	no	bacterium	MRSA	0	—	=
26	IC_50_ (μM)	no	no	cell line	Hep2	0	NA	=
27	IC_50_ (μM)	no	no	cell line	HT29	0	NA	=
28	IC_50_ (μM)	no	no	cell line	Human Vero cells	0	NA	=
29	IC_50_ (μM)	no	no	cell line	Human Vero cells	0	NC	<>
30	IC_50_ (μM)	no	no	cell line	LMM3	0	NA	=
31	IC_50_ (μM)	no	no	cell line	PTP	0	2	>
32	IC_50 _(μM)	no	no	fungus	*C. albicans*	0	—	=
33	IC_50_ (μM)	no	no	fungus	*C. neoformans*	0	—	=
34	IC_50_ (μM)	no	no	parasite	*L. mexicana*	0	2	>
35	IC_50_ (μM)	no	no	parasite	*P. falciparum*	0	2	>
36	IC_50_ (μM)	no	no	parasite	*P. falciparum D6*	0	NA	=
37	IC_50_ (μM)	no	no	parasite	*P. falciparum* W2	0	NA	=
38	IC_50_E-9 (M)	GSK-3	nd	enzyme	no	0	2	>
39	IC_90_ (μg/mL)	no	no	parasite	*L. donovani*	0	NA	=
40	MIC (μg/mL)	no	no	bacterium	*M. tuberculosis* (H37Rv)	0	NA	=
41	pIC_50_	GSK-3	beta	enzyme	no	0	0	=
42	pIC_50_	GSK-3	nd	enzyme	no	0	0	=
43	IC_50_ (μM)	no	no	no	Cell Efficacy	0	2	>

The problem in this type of organization of raw data is that the descriptor values are compound constants. Consequently, if these latter and LDA are based only on these values, they will necessarily fail when we change OG values. An inconvenient in this regard occurs if we pretend to use the model for a real enzyme, since we have only one unspecific prediction and we need 43 specific probabilities, one confirming the real class and 42 giving low probabilities for the other CAC_q_. We can solve this problem introducing variables characteristic of each CAC class referred on the CAC_q_ but without giving information in the input about the real CAC class of the protein. To this end, we used the average value of each descriptor for all enzymes that belonged to the same CAC class. We also calculated the deviation of all the descriptors from the respective group indicated in CAC_q_. Altogether, we have then (63 topological descriptor values) + (63 topological descriptor values average values for CAC class) + (63 topological descriptor values deviations values for CAC class average) = 189 input variables. It is of major importance to understand that we never used as input CAC_q_, so the model only includes as input the physicochemical-topological descriptor values for the protein entry and the average and deviations of these values from the CAC_q_, which is not necessarily the real CAC class. The general formula for this class of LDA model is shown below (see **Equation 2**), where S(E) is not the probability but a real valued score that predicts the propensity of a compound to act as an inhibitor of a given class:

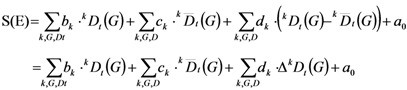
(2)


In a compact notation we write ^k^D_t_(G), where D_t_ is the type of descriptor; G is the types of subgraphs studied in the molecular connectivity G = path (*p*), clusters (*C*), path-clusters (*pC*) and chains (rings) (*Ch*).

LDA forward stepwise analysis was carried out for variable selection to build up the models [68]. All the variables included in the model were standardized in order to bring them onto the same scale. Subsequently, a standardized linear discriminant equation that allows comparison of their coefficients was obtained [69]. The square of Canonical regression coefficient (Rc) and Wilk’s statistics (U) were examined in order to assess the discriminatory power of the model (U = 0 perfect discrimination, being 0 < U < 1); the separation of the two groups was statistically verified by the Fisher ratio (F) test with an error level p < 0.05.

### 3.3. Data Set

The data set was conformed to a set of marketed and/or reported drugs/receptor pairs where affinity/non-affinity of drugs with the receptors was established taking into consideration the IC50, *k*i, *pk*i, values. In consequence, we managed to collect 1,192 examples of active compounds in different CAC_q_. In addition, we used a negative control series of 2,178 cases of non-active compounds in different CAC_q_. In the two data sets used, there were the following training series: 474 active compounds plus 896 non-active compounds (1,370 in total), predicting series: 296 + 1,164 = 1,460 in total. Due to space constraints the names or codes for all compounds are listed in supplementary material **SM1** in the Supporting Information, as well as the references consulted to compile the data in this table. This series is composed at random by the most representative families of GSK-3 inhibitors taken from the literature (supplementary material **SM2**). The remaining compounds were a heterogeneous series of inactive compounds, including members of the aforementioned families and compounds included in the Merck index [70].

## 4. Conclusions

In this work we have shown that the ModesLab methodology using topological indices can be considered a good alternative for developing GSK-3 inhibitors in a fast and efficient way with respect to other methods of the literature. This approach is able to correctly classify the GSK-3 inhibitory activity of compounds with different structural patterns.
